# *Dendrobium huoshanense* C. Z. Tang and S. J. Cheng can be prepared as a food with the ability to prevent and treat hyperuricaemia

**DOI:** 10.3389/fnut.2025.1518014

**Published:** 2025-03-11

**Authors:** Qing Hao, Lin Jiang, Jun Ma, Huikai Wang, Ying Liu, Qichang Xu, Songze Li, Song Han, Qiusheng Zheng, Xiangcheng Fan, Jichun Han

**Affiliations:** ^1^College of Traditional Chinese Medicine, Binzhou Medical University, Yantai, China; ^2^Binzhou Medical University Affiliated Traditional Chinese Medicine Hospital, Binzhou Medical University, Binzhou, China; ^3^Anhui Hushengji Biotechnology Co., Ltd., Hefei, China; ^4^Department of Pharmacy, Center for X Medicine, The Fourth Affiliated Hospital of School of Medicine, and International School of Medicine, International Institutes of Medicine, Zhejiang University, Yiwu, China; ^5^Center for Innovative Traditional Chinese Medicine Target and New Drug Research, International Institutes of Medicine, Zhejiang University, Yiwu, China

**Keywords:** *Dendrobium huoshanense* C. Z. Tang and S. J. Cheng, hyperuricemia, homology-of-medicine-and-food, untargeted metabolomics, functional food

## Abstract

**Background:**

Hyperuricemia (HUA) is the presence of excessive uric acid (UA) in blood, which leads to an increased risk of chronic kidney disease and gout. There are about 120 million hyperuricemia patients in China, which has surpassed diabetes as the second largest chronic disease.

**Objective:**

The aim of the present study was to investigate the hypouricemic effects of *Dendrobium huoshanense* C. Z. Tang and S. J. Cheng (DH), and provide a basis for its development into anti HUA products.

**Methods:**

This study established a mouse HUA model by gavage of potassium oxonate (PIO) and hypoxanthine (HX), and treated with DH to investigate the therapeutic effect of DH on hyperuricemia. Use a biochemical assay kit to detect changes in the levels of UA, creatinine (Cr), and urea nitrogen (BUN) in mouse serum; Use ELISA kit to detect the activity of xanthine oxidase (XOD) in serum; Untargeted Metabolomics analysis was performed on the serum of each group of mice using liquid chromatography-mass spectrometry. This study recruited 23 HUA volunteers, volunteers drank 0.5 g DH daily for four consecutive weeks, with serum UA levels measured once a week.

**Results:**

Animal experiments have shown that DH has therapeutic effects on HUA, mainly manifested as: DH effectively reduces the levels of UA, Cr, and BUN in the serum of HUA mice, lowers XOD activity in the serum, and alleviates kidney tissue and glomerular damage. Metabolomics analysis showed that there were 306 significant differences in metabolites between the Sham group, HUA model group, and DH group. Pathway analysis of these differential metabolites revealed that they were mainly involved in pyrimidine metabolism, histidine metabolism, and riboflavin metabolism. Clinical research results show that after drinking DH, UA levels in HUA volunteers significantly decreased, and most HUA volunteers' UA levels decreased to normal levels.

**Conclusion:**

DH has the effect of preventing and treating hyperuricemia, and it belongs to the same class of traditional Chinese medicine as medicine and food, with extremely low toxicity and high safety. Therefore, DH is suitable for preparation as a product for preventing and treating HUA in functional food and other products.

## Graphical Abstract

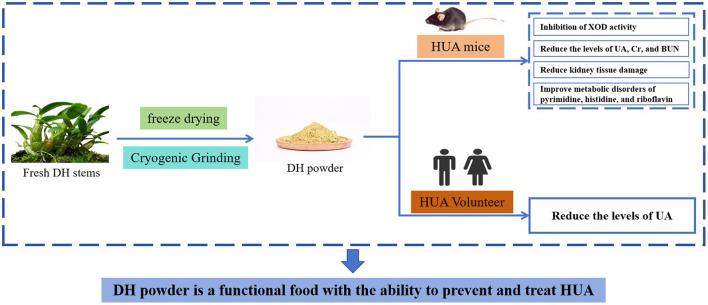

## 1 Introduction

Hyperuricemia (HUA) is a common metabolic disease in clinical practice, caused by disorders in purine metabolism in the body. Under a normal purine diet, fasting blood uric acid (UA) levels increase twice on different days, with males exceeding 420 μmol/L and females exceeding 360 μmol/L, indicating HUA (1). HUA is closely related to the occurrence and development of gout, kidney stones, cardiovascular and cerebrovascular diseases, type 2 diabetes, metabolic syndrome and other diseases, especially gout, HUA is the main cause of gout (2–4). In 2020, the total number of HUA patients worldwide exceeded 1.1 billion, and it is expected to reach 1.42 billion by 2030, and the patients have become younger, which is of concern, so HUA has emerged as a public health challenge (5, 6). The overall prevalence rate of HUA in China is 13.3%, and the number of patients is about 177 million. It has become the second largest metabolic disease after diabetes, posing a serious threat to the life and health of the Chinese people (7, 8). Therefore, it is urgently needed to develop safe and effective methods for HUA prevention and control.

At present, the main methods for treating HUA are to reduce serum UA levels through some drugs, including drugs that inhibit UA synthesis, promote UA excretion, and promote UA decomposition (9). Xanthine oxidase (XOD) inhibitors are common drugs that inhibit UA synthesis, such as allopurinol and febuxostat, which inhibit XOD activity, reduce the oxidation of xanthine and hypoxanthine to UA and xanthine in the human body, and lower serum UA levels. Although XOD inhibitors can reduce uric acid concentration, long-term use can cause some adverse reactions, such as allergic reactions and liver and kidney damage (10). Analyzing the pathogenesis of HUA, it can be found that the majority of HUA patients develop the disease mainly due to poor excretion of UA. Therefore, drugs that promote UA excretion are more suitable for the treatment of HUA patients to a certain extent. At present, the commonly used drugs for promoting UA excretion in clinical practice are allopurinol drugs, such as benzbromarone tablets and febuxostat tablets (11). Benzobromarone and other drugs that promote UA excretion can effectively inhibit the reabsorption of UA by renal tubules, rapidly reduce UA concentration, and accelerate UA excretion rate. However, long-term use of such drugs can also lead to adverse reactions in patients, mainly manifested in their impact on the human liver and causing allergic reactions such as itching (12, 13). Uric acid oxidase is a commonly used drug that promotes the breakdown of UA, which is divided into non recombinant uric acid oxidase and recombinant uric acid oxidase drugs. Urinase is an oxidase that promotes the oxidative degradation of UA, converting it into UA and reducing its absorption by the renal tubules, thereby facilitating its excretion from the body. At present, uricase drugs are commonly used in clinical treatment, which have good therapeutic effects. However, there are some limitations, such as high prices and susceptibility to allergic reactions (14, 15). The above indicates that although existing drugs can effectively reduce UA, there are some shortcomings, such as long-term use causing liver and kidney damage and allergic reactions, and being expensive. Based on the defects and deficiencies of existing treatment, it is urgent to seek a strategy with obvious effect, stability, safety, and convenience for HUA.

In addition to medicine, some foods also have the effect of treating diseases. In China, some plants can be used as food for people to eat or as medicine to treat some diseases. These herbs are collectively referred to as homology-of-medicine-and-food (HMF), such as *Codonopsis pilosula, Cistanche deserticola*, and *Ziziphus jujuba* seeds (16). Compared to drugs, HMF has extremely low toxicity and long-term use will not cause toxic side effects. This is very suitable for treating chronic diseases and also for long-term consumption to prevent some diseases. This is very suitable for treating chronic diseases and also for long-term consumption to prevent some diseases (17). Therefore, searching for some HMFs to prevent and treat HUA may be a feasible strategy.

*Dendrobium huoshanense* C. Z. Tang and S. J. Cheng (DH) is a unique Chinese herbal medicine that belongs to HMF. It has various biological activities, such as antioxidant and anti-inflammatory effects. Our previous research found that DH has a good lipid lowering effect and can reduce atherosclerosis induced by high cholesterol diet (18, 19). At present, there is no research report on the effect of DH on reducing UA. Therefore, this study aims to investigate whether DH has a reducing effect on UA and develop it into a product for the prevention and control of HUA.

## 2 Materials and methods

### 2.1 Chemicals and materials

DH was purchased from Anhui Hushengji Biotechnology Co., Ltd. (Hefei, Anhui Province, China). Micro Uric Acid (UA) Content Assay Kit, Urea Nitrogen (BUN) Content Assay Kit, Creatinine (Cr) Content Assay Kit, and Xanthine Oxidase (XOD) Activity Assay Kit was purchased from Beijing Solarbio Science and Technology Co.,Ltd. (Beijing, China). Potassium Oxonate (abbreviation: PIO, purity ≥99.0%) and Hypoxanthine (abbreviation: HX, purity ≥99.0%) were purchased from Beijing Solarbio Science and Technology Co., Ltd. (Beijing, China). Non targeted metabolomics detection related reagents are provided by Solaibao. Untargeted Metabolomics detection related reagents are provided by Shenzhen Weike Meng Technology Group Co., Ltd. (Shenzhen, China).

### 2.2 Preparation of DH powder

To preserve more active ingredients of DH, fresh DH stems were selected as raw materials in this study and prepared into powder at low temperature. The specific operation is as follows: pick fresh DH stems aged 3–5, clean them, and air dry them. Place the dried DH stems in a vacuum freeze dryer at a temperature of around −60 to −80°C for 4 h. Dry fresh DH stems were placed in a frozen low-temperature ultrafine grinder for low-temperature grinding into powder, and sieved through a 200 mesh sieve to obtain fresh DH powder, which is the research sample of this study (Graphical Abstract).

### 2.3 Animals and ethics statement

A total of 50 adult male C57BL/6 mices (4 weeks old) were ordered for this study. All animals were purchased from Sibeifu (Beijing) Biotechnology Co., Ltd. License Number: SCXK(jing)2019-0010. All animals were kept in steel rodent cages, and the animal room temperature was controlled at 22 ± 2°C. The humidity was controlled at 60%−80%, with a 12 h light:dark cycle (light period: 07:00-19:00; dark period: 19:00–07:00). The animal use protocol listed below was reviewed and approved by the Binzhou Medical University Institutional Animal Care and Use Committee (2023-376).

### 2.4 Mice HUA model

A mice HUA model was constructed by gavage of PIO (300 mg/kg) + HX (500 mg/kg). The weight of the mice is 15 ± 2 g. To ensure consistent gavage volume and reduce errors, the gavage volume of PIO and HX was designed to be 0.1 ml. PIO was administered orally with a volume of 0.1 ml for 4 weeks, and PIO solution was prepared by adding 450 mg of PIO and 10 ml of 0.5% CMC-Na. Administer 0.1 ml of HX by gavage for 4 weeks, and prepare HX solution: 750 mg of HX+10 ml of 0.5% CMC-Na.

### 2.5 Design of animal experiments group

**Figure 1:**
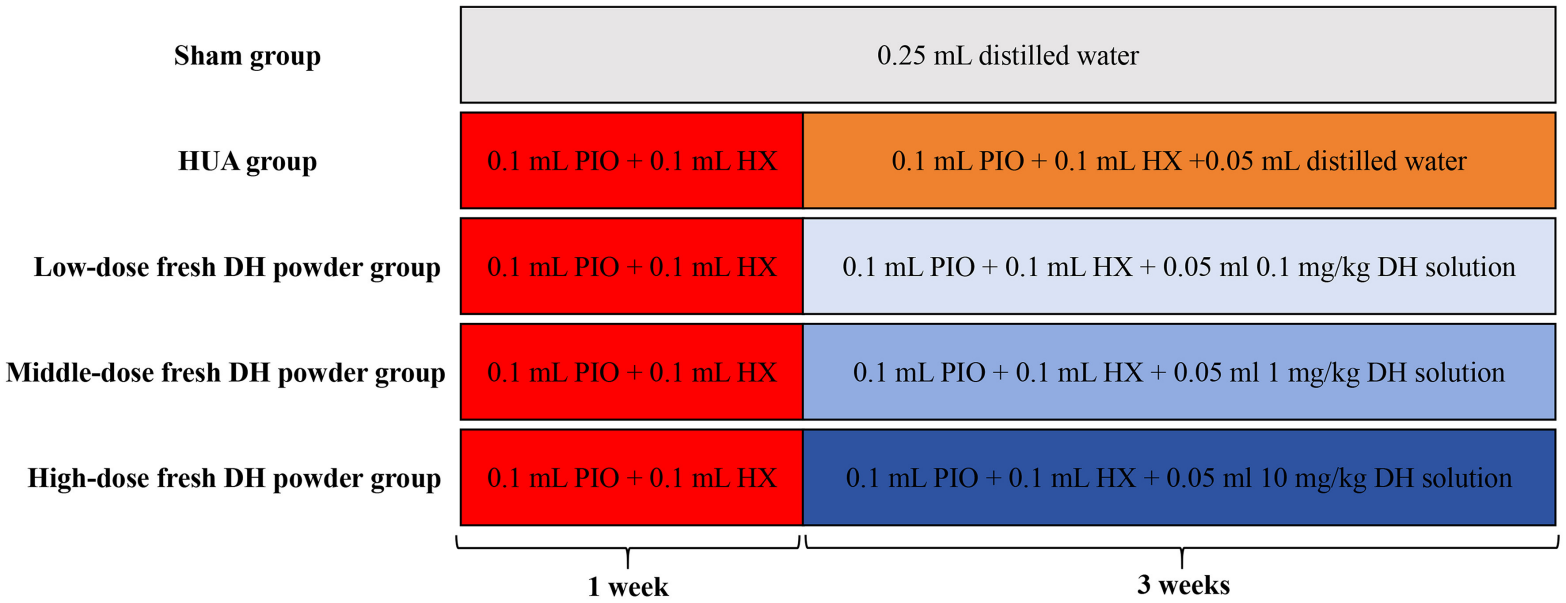
Grouping processing flowchart.

Fifty adult male C57BL/6 mice were randomly divided into five groups: sham group, HUA model group, low-dose fresh DH powder group (0.1 mg/kg), middle dose fresh DH powder group (1 mg/kg), and high-dose fresh DH powder group (10 mg/kg). In order to ensure consistent gavage volume and reduce errors, the gavage volume of fresh DH powder is designed to be 0.05 ml. 0.1 mg/kg fresh DH powder solution preparation: 3 mg fresh DH powder + 100 ml distilled water. 1 mg/kg fresh DH powder solution preparation: 30 mg fresh DH powder + 100 ml distilled water. 10 mg/kg fresh DH powder solution preparation: 300 mg fresh DH powder + 100 ml distilled water. The treatment for each experimental group is as follows (Figure 1): Sham group: 0.25 ml distilled water was orally administered for 4 weeks. HUA model group: administer 0.1 ml PIO solution + 0.1 ml HX solution by gavage. After 1 week, start gavage of 0.1 ml PIO solution+0.1 ml HX solution + 0.05 ml distilled water for 3 weeks. Low dose fresh DH powder group: gavage of 0.1 ml PIO solution + 0.1 ml HX solution. After 1 week, start gavage of 0.1 ml PIO solution + 0.1 ml HX solution + 0.05 ml 0.1 mg/kg fresh DH powder solution for 3 weeks. Middle dose fresh DH powder group: gavage of 0.1 ml PIO solution + 0.1 ml HX solution. After 1 week, start gavage of 0.1 ml PIO solution + 0.1 ml HX solution + 0.05 ml 1 mg/kg fresh DH powder solution for 3 weeks. High dose fresh DH powder group: gavage of 0.1 ml PIO solution + 0.1 ml HX solution. After 1 week, start gavage of 0.1 ml PIO solution + 0.1 ml HX solution + 0.05 ml 10 mg/kg fresh DH powder solution for 3 weeks.

### 2.6 Biochemical index detection

After the modeling is completed, 1 ml of blood is taken from the mouse eyeball, left at room temperature for 2 h, centrifuged (3,500 rpm) for 10 min, collected and packaged, and stored at −20°C for future use. Strictly follow the instructions of the UA, Cr, BUN, and XOD test kits to detect the levels of UA, Cr, BUN, and XOD in serum.

### 2.7 Detecting pathological damage to the kidneys and glomeruli

After blood collection, C57BL/6 mice were euthanized by cervical dislocation method, and their livers were removed. The livers were washed with pre cooled physiological saline and dried with filter paper. The livers were then fixed with 4% neutral formaldehyde. HE stained sections were used to observe pathological damage in renal tissue, while PAS stained sections were used to observe pathological damage in glomeruli.

### 2.8 Serum untargeted metabolomics detection

Non targeted metabolomics analysis was performed on the serum of each group of mice using liquid chromatography-mass spectrometry. After the modeling was completed, 1 ml of blood was collected from the mouse eyeball, left at room temperature for 2 h, centrifuged at 3,500 rpm for 10 min, and 150 μl of serum was collected. The serum was then sent to Weike Meng Technology Company for untargeted metabolomics analysis.

### 2.9 Case collection

Case source: it is expected to select 80 patients with hyperuricemia admitted to the Affiliated Hospital of Binzhou Medical College from March 2023 to October 2023 as the research group, including 40 males and 40 females, aged between 40 and 65 years old.

Case selection criteria: developed based on the ACR gout classification criteria of 1977 and the ACR/EULAR gout classification criteria of 2015.

Inclusion criteria: ① Meets the diagnostic criteria for hyperuricemia, which means that the diagnostic criteria for hyperuricemia are blood uric acid levels measured twice on non same day fasting, with males >420 μmol/L and females >360 μmol/L; ② Age between 40 and 65 years old, gender not limited; ③ Acute onset, with a course of disease within 14 days; ④ No gout symptoms have appeared yet; ⑤ All participants provided informed consent and signed an informed consent form.

Exclusion criteria: not taking diuretics or medications to lower uric acid levels within 3 months; Patients with acute and chronic infection, hypertension, coronary heart disease, malignant tumor, liver and kidney diseases, diabetes and blood system diseases; Individuals with abnormal liver and kidney function or gout; Pregnant and lactating women.

Case dropout criteria: ① Those who experience serious adverse events or complications and are not suitable for continued treatment; ② Poor compliance of subjects, using medication that does not reach 80% of the prescribed amount or exceeds 120% of the prescribed amount; ③ Patients who voluntarily withdraw or are lost to follow-up during the treatment process; ④ Those who have not completed the entire course of treatment and have affected the efficacy or safety assessment; ⑤ Incomplete information affects the validity and safety of the judgment.

Case dropout treatment: ① After the subject falls off, the researcher should try to contact the subject as much as possible, inquire about the reasons, and complete the evaluation items as much as possible; ② The dropout cases are all required for the full analysis set statistics, and dropout patients do not need to be supplemented separately.

Termination test criteria: ① Those who have experienced serious adverse events; ② During the course of the disease, the condition worsened and ineffective cases were treated; ③ Serious deviations occurred during the implementation of clinical trial protocols; ④ The subjects requested to withdraw during the clinical trial process.

### 2.10 DH efficacy testing

A total of 23 HUA volunteers were selected for this study, 15 males and 8 females. Volunteers drank fresh DH powder continuously for 4 weeks, with a daily intake of 0.5 g. Perform weekly testing of serum UA levels in HUA volunteers, and retest serum UA levels after discontinuing fresh DH powder for 1 month. The clinical trial has been approved by the Medical Ethics Committee of Binzhou Traditional Chinese Medicine Hospital (2024-023).

### 2.11 Safety evaluation criteria

Referring to the “Guidelines for Clinical Research of Traditional Chinese Medicine New Drugs (Trial)” in 2002, the main observation is on patients' digestive disorders and abdominal pain before and after treatment, as well as other adverse reactions such as gastrointestinal discomfort, bloating, vomiting, diarrhea, bloody stools, and black stools, and safety indicators such as liver and kidney function. The degree is classified as follows: ① None: no adverse reactions; Mild: ② adverse reactions are mild, short-lived, and tolerable; ③ Moderate: the reaction is severe, stop taking the medication, and it can be relieved without treatment; ④ Severe: severe reaction, treatment terminated, symptomatic treatment.

### 2.12 Statistical analysis

Data are presented as the mean ± standard deviation from at least six independent experiments. Statistical differences were determined by using a Student's *t*-test, where *P* < 0.05 was considered to be statistically significant. The analyses were performed by using the Statistical Program for Social Sciences Software (International Business Machines Corporation, New York, USA).

## 3 Results

### 3.1 DH reduces HUA in mice

**Figure 2:**
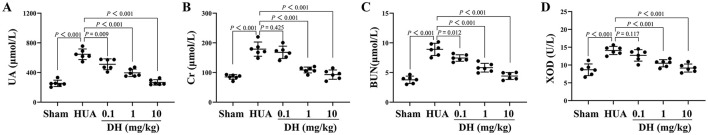
DH significantly reduced the levels of UA **(A)**, Cr **(B)**, BUN **(C)**, and XOD activity **(D)** in the serum of HUA mice.

As shown in Figure 2, the serum UA level in the sham group of mice was 253.24 ± 39.37 μmol/L, while the UA level in the HUA group significantly increased to 646.10 ± 65.22 μmol/L. Compared with the HUA group, DH treatment significantly reduced UA levels, with 0.1 mg/kg DH reducing UA to 511.25 ± 66.59 μmol/L, 1 mg/kg DH reducing UA to 395.95 ± 46.33 μmol/L, and 10 mg/kg DH reducing UA to 266.81 ± 33.05 μmol/L. The serum Cr level in the sham group of mice was 85.08 ± 7.31 μmol/L, while the Cr level in the HUA group significantly increased to 178.82 ± 21.85 μmol/L. Compared with the HUA group, DH treatment significantly reduced Cr levels, with 0.1 mg/kg DH reducing Cr to 168.07 ± 18.93 μmol/L, 1 mg/kg DH reducing Cr to 108.54 ± 9.08 μmol/L, and 10 mg/kg DH reducing Cr to 92.86 ± 14.43 μmol/L. The serum BUN level in the sham group of mice was 3.77 ± 0.56 μmol/L, while the BUN level in the HUA group significantly increased to 8.90 ± 0.94 μmol/L. Compared with the HUA group, DH treatment significantly reduced BUN levels, with 0.1 mg/kg DH reducing BUN to 7.44 ± 0.50 μmol/L, 1 mg/kg DH reducing BUN to 5.82 ± 0.67 μmol/L, and 10 mg/kg DH reducing BUN to 4.41 ± 0.53 μmol/L. The serum XOD activity in the sham group of mice was 8.67 ± 1.49 U/L, while the XOD activity in the HUA group significantly increased to 14.10 ± 1.05 U/L. Compared with the HUA group, DH treatment significantly reduced XOD activity, with 0.1 mg/kg DH reducing XOD to 12.72 ± 1.47 U/L, 1 mg/kg DH reducing XOD to 10.50 ± 0.92 U/L, and 10 mg/kg DH reducing XOD to 9.15 ± 1.03 U/L.

### 3.2 DH alleviates renal tissue damage in HUA mice

**Figure 3:**
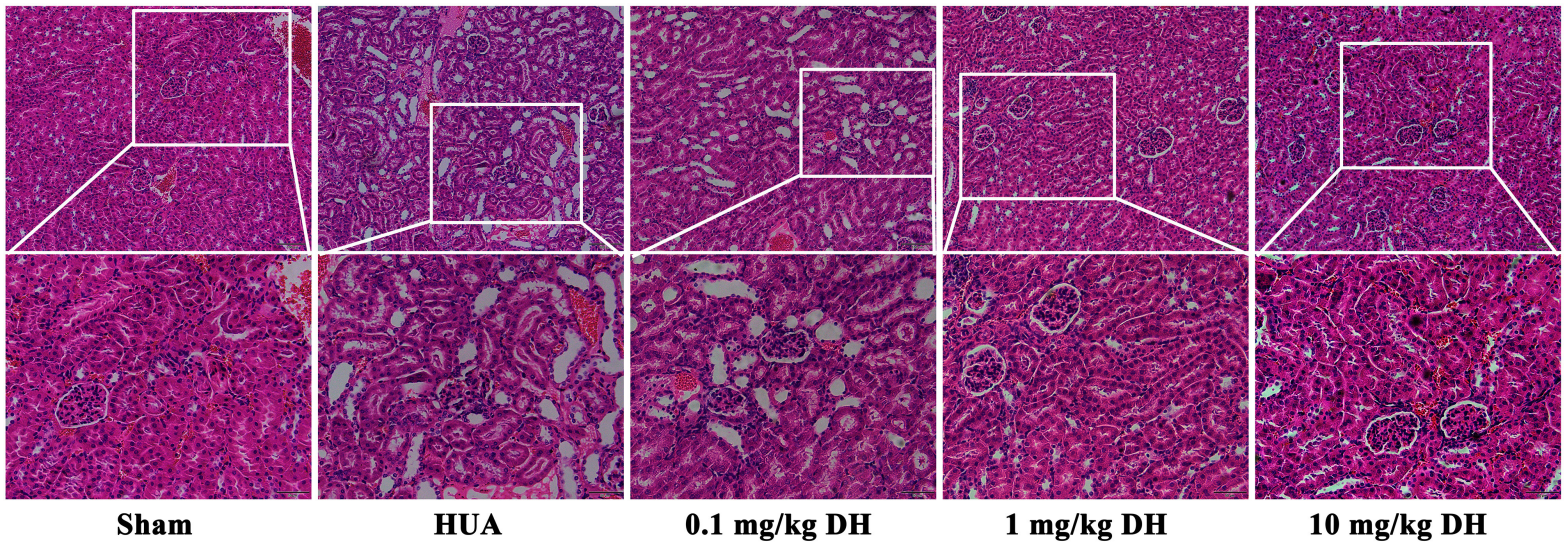
HE staining results showed that DH reduced renal tissue damage in HUA mice.

As shown in Figure 3, the HE results showed that the renal tissue cells of the sham group mice were arranged neatly, with clear and full glomerular and tubular structures, uniform staining of tubular epithelial cells, and normal morphology. In the renal tissue of HUA group mice, glomerular deformation and atrophy were observed, renal tubules were dilated, and a large number of vacuolar changes were observed in the cells. Compared with the HUA group, the low-dose DH group showed similar kidney damage in mice; the renal tissue cells of mice in the middle and high dose DH groups were arranged neatly, with clear and full glomerular and tubular structures, uniform staining of tubular epithelial cells, and normal morphology.

### 3.3 DH reduces glomerular injury in HUA mice

**Figure 4:**
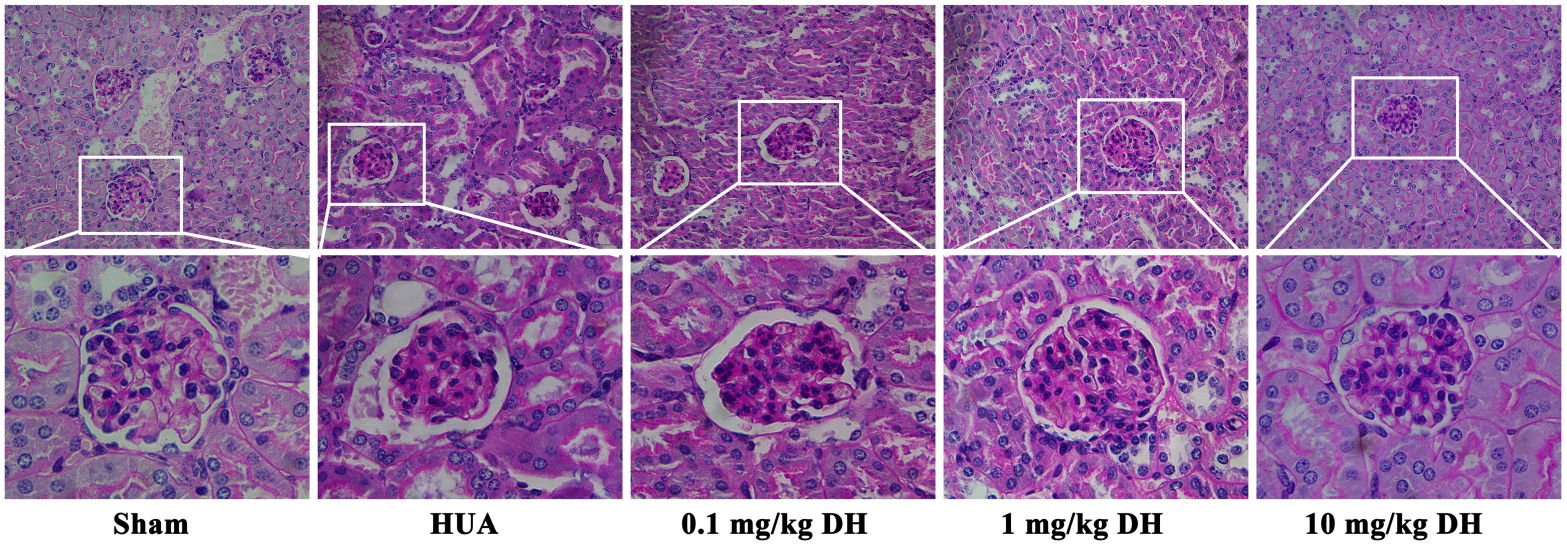
PAS staining results showed that DH alleviated glomerular injury in HUA mice.

As shown in Figure 4, the PAS staining results showed that the glomerular structure of the sham group mice was clear, full, and had normal morphology. The glomeruli of mice in the HUA group showed thickening of the basement membrane. Compared with the HUA group, the low-dose DH group mice also showed thickening of the basement membrane in their glomeruli, while the middle and high-dose DH group mice had clear and plump glomeruli structures and normal morphology.

### 3.4 The effect of DH on the metabolic level of HUA mice

**Figure 5:**
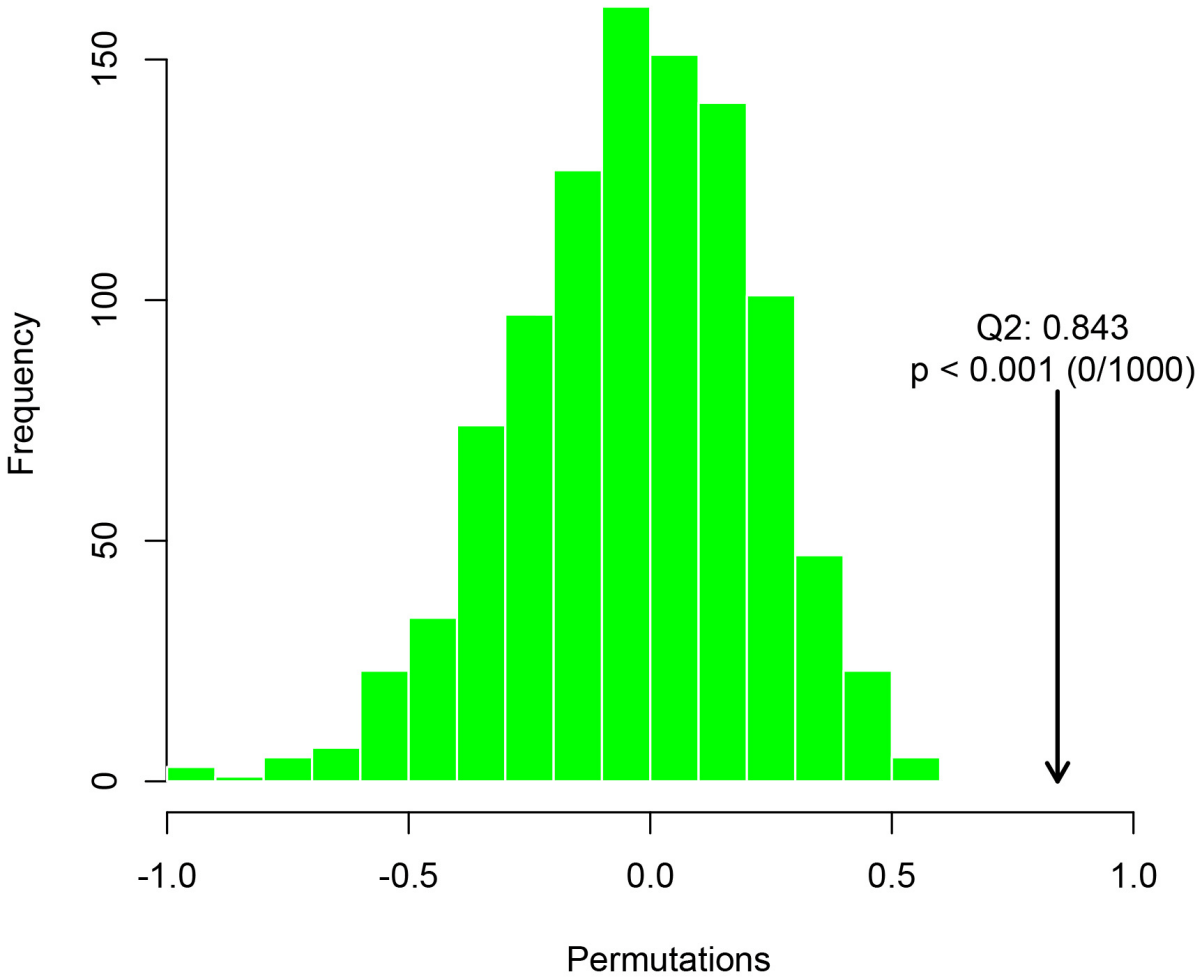
Distribution and p-value of test statistic (Q2) for OPLS-DA permutation test.

**Figure 6:**
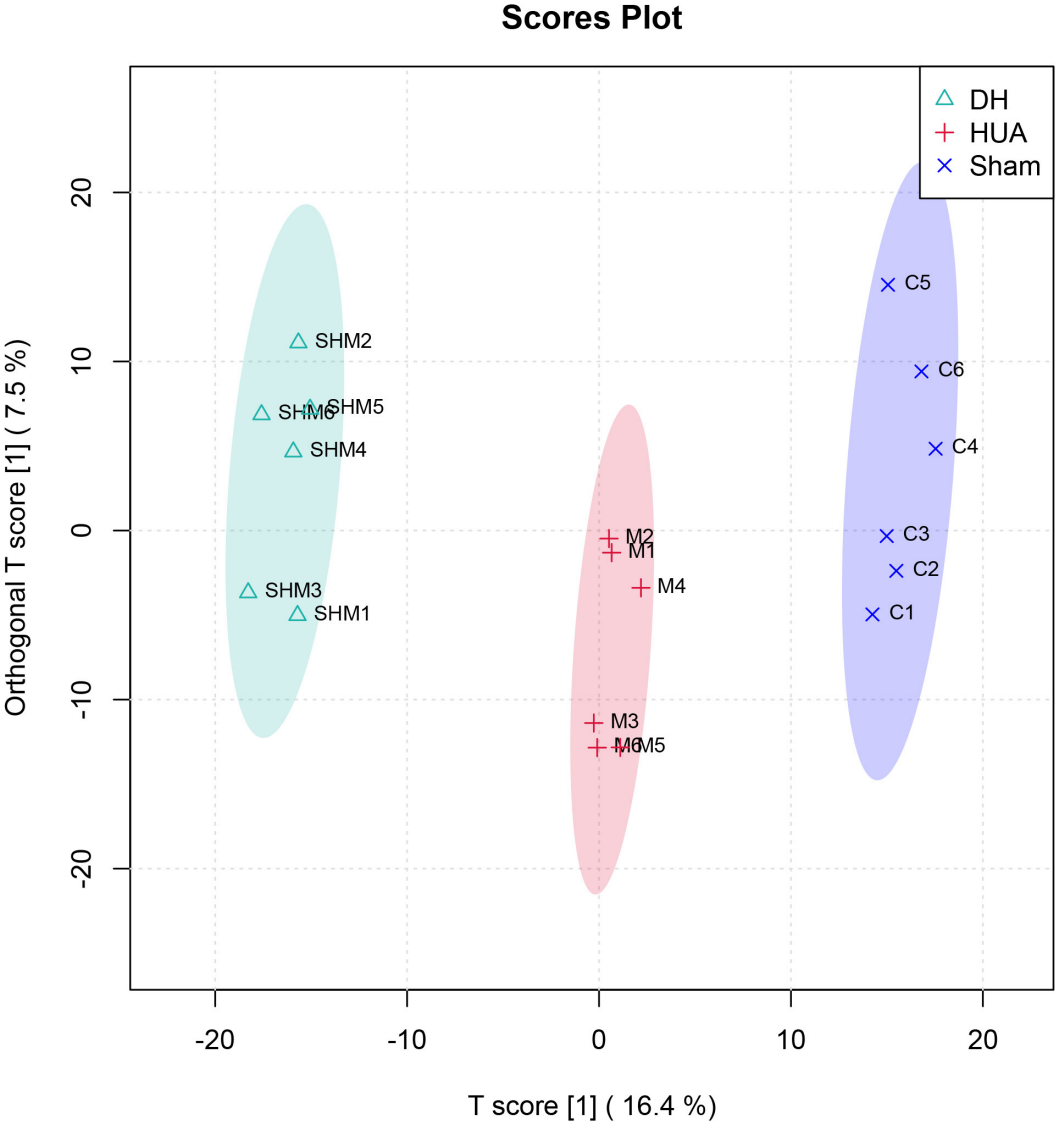
OPLS-DA point cloud map.

**Figure 7:**
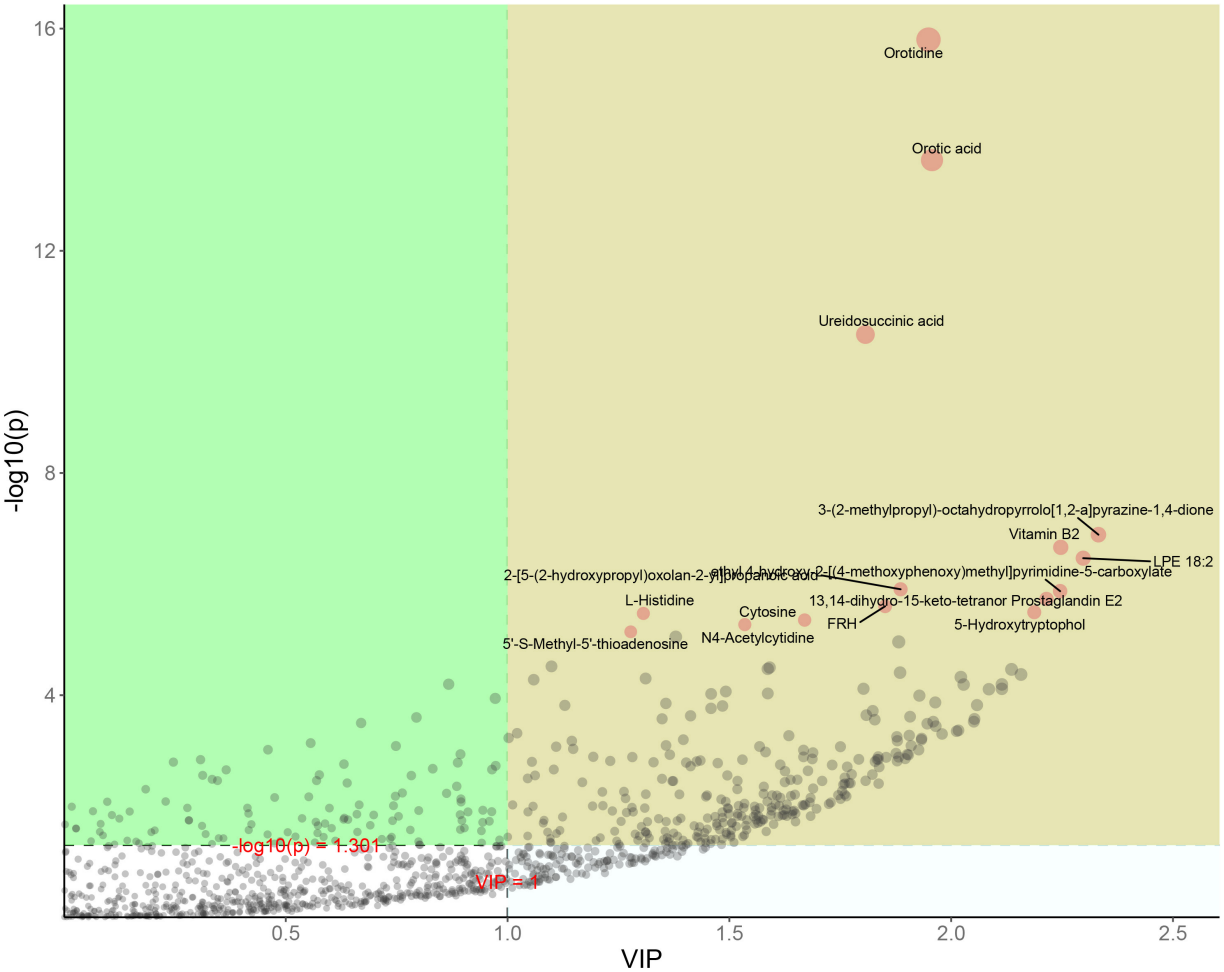
OPLS-DA metabolite importance map.

**Figure 8:**
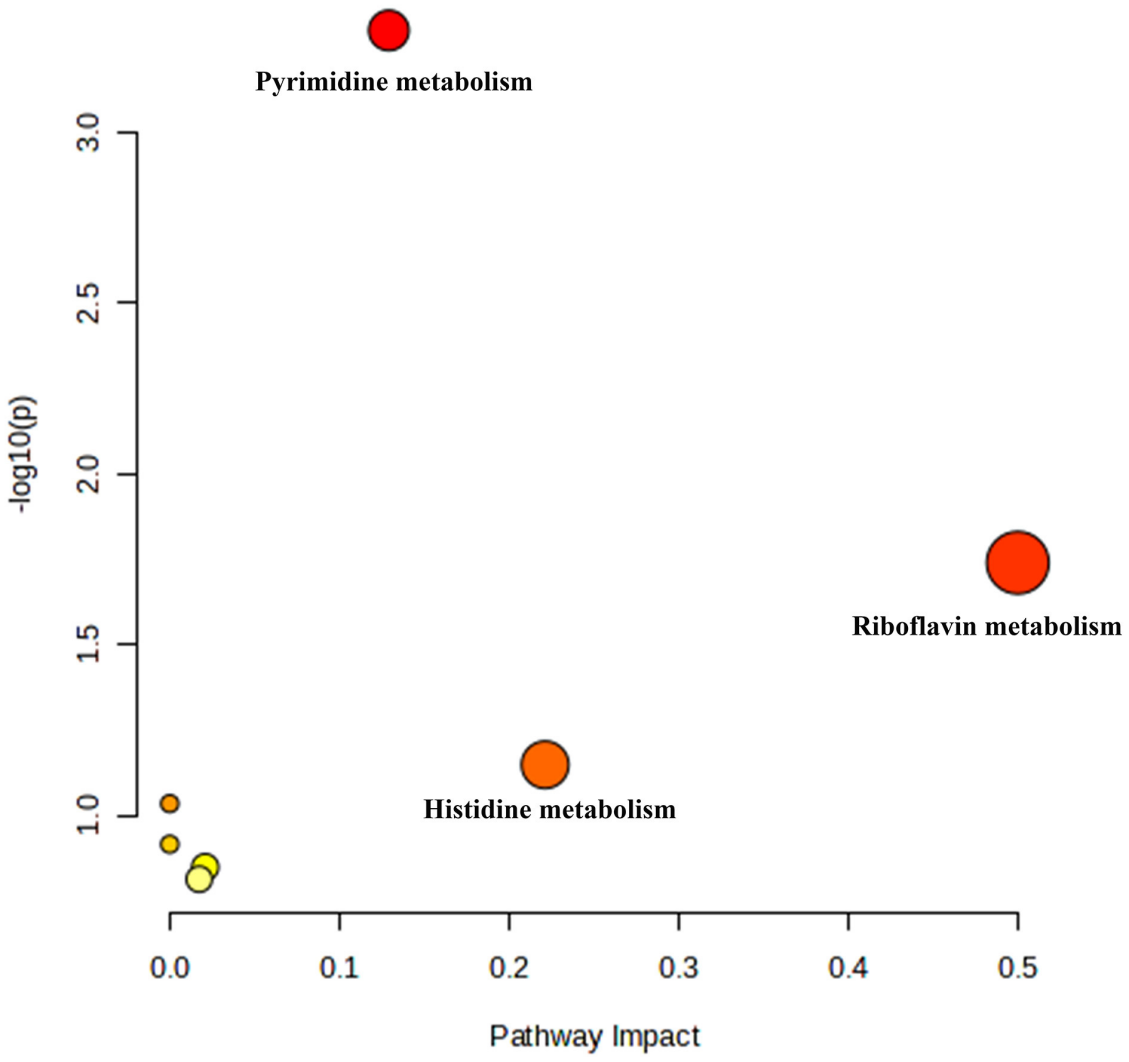
MetaboAnalyst 5.0 performed pathway analysis on differential metabolites, resulting in a bubble chart.

The high-dose treatment had the best therapeutic effect among the three DH treatment groups, so metabolomics analysis was conducted using sham group, HUA group, and high-dose DH group. Using OPLS-DA and Q2 as the test statistic, the random distribution of Q2 is obtained by permutation method. As shown in Figure 5, Q2 > 0.5, *P* < 0.5. The model is appropriate and has significant predictive ability, indicating that there should be significant differences in metabolites between groups. The OPLS-DA point cloud map results showed that the data between the sham group, HUA group, and DH group were separated, while the data within each group were closely aligned, indicating significant changes in serum metabolite levels among the three groups of mice (Figure 6). According to the variable importance in the projection (VIP) threshold generated by OPLS-DA processing, metabolites were screened and set to VIP >1. The *P*-value for significantly different metabolites is set to 0.05. According to the above criteria, this study identified and selected the top 15 metabolites with significant differences through secondary mass spectrometry. HUA can cause an increase or decrease in the content of some metabolites, while DH can reverse the changes in these metabolites caused by HUA (Figure 7 and Table 1). Through MetaboAnalyst 5.0 (https://www.metaboanalyst.ca/) was used to perform pathway analysis on differential metabolites, and the results are shown in Figure 8. The above metabolites are involved in pyrimidine metabolism, histidine metabolism, and riboflavin metabolism.

**Table 1:** Top 15 differential metabolite mass spectrometry data.

**Metabolite**	**RT/min**	***m*/*z***	**VIP**	**Sham**	**HUA**	**DH**
Orotidine	1.36	287.05	2.03	78.79	42.10^#^	46.36^*^
L-dihydroorotic acid	8.91	157.03	2.03	78.68	41.13^#^	45.95^*^
Ureidosuccinic acid	1.37	175.04	1.88	75.27	49.87^#^	47.90
3-(2-methylpropyl)-octahydropyrrolo [1,2-a]pyrazine-1,4-dione	5.56	211.14	2.31	72.55	43.44^#^	44.43
Vitamin B2	9.33	375.13	2.29	77.22	12.24^#^	34.58^*^
LPE 18:2	8.83	476.28	2.30	75.72	24.13^#^	38.44^*^
Ethyl4-hydroxy-2-[(4-methoxyphenoxy)methyl] pyrimidine-5-carboxylate	5.57	305.11	2.21	67.57	52.07^#^	45.73^*^
13,14-dihydro-15-keto-tetranor Prostaglandin E2	6.57	297.17	2.22	75.39	10.21^#^	33.11^*^
5-Hydroxytryptophol	5.09	178.09	2.17	65.75	54.05^#^	45.76^*^
2-[5-(2-hydroxypropyl)oxolan-2-yl]propanoic acid	1.18	185.11	1.91	73.20	34.67^#^	41.40^*^
L-histidine	1.23	156.08	1.33	60.20	67.96^#^	48.80^*^
Cytosine	1.38	112.05	1.72	69.03	45.45^#^	43.82
FRH	9.22	459.25	1.88	72.27	35.39^#^	41.31^*^
5′-S-Methyl-5′-thioadenosine	5.10	298.10	1.39	61.36	63.52	47.60^*^
N4-acetylcytidine	4.84	286.10	1.61	66.67	51.96^#^	45.34^*^

DH represents high-dose group.

^#^P < 0.05 compared to the sham group.

^*^P < 0.05 compared to the HUA group.

### 3.5 DH reduces HA levels in HUA patients

**Figure 9:**
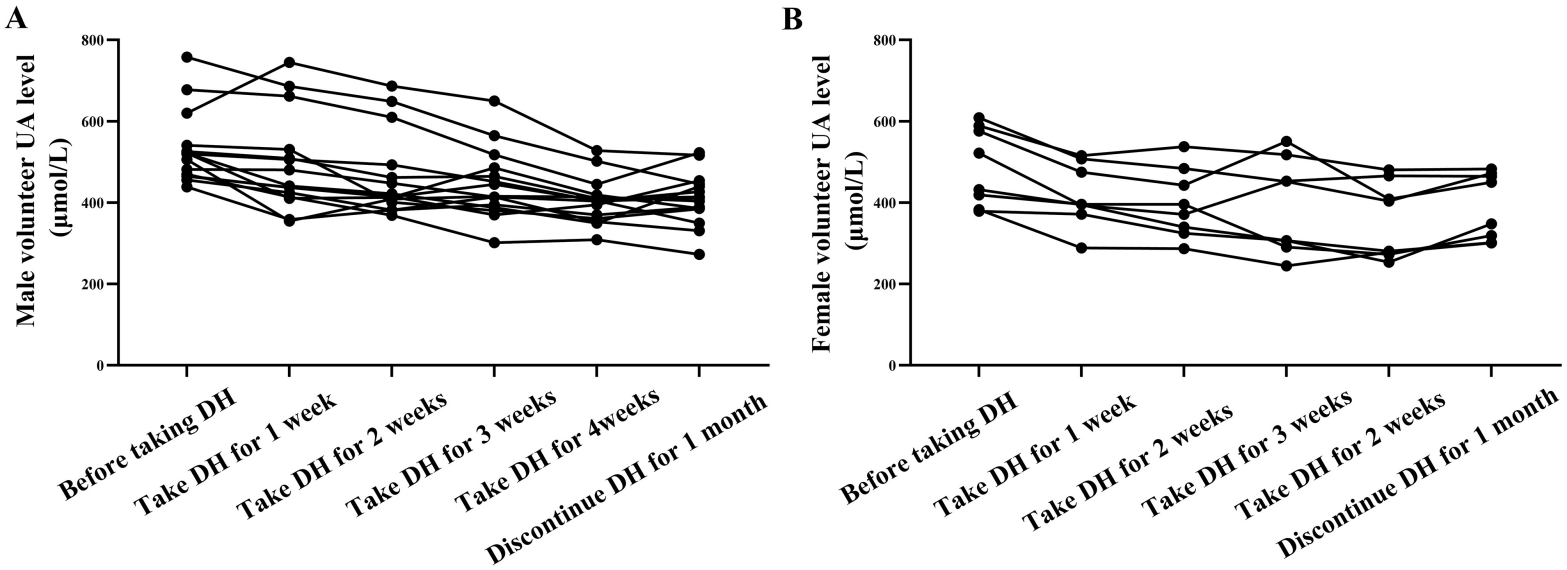
DH reduces UA levels in male **(A)** and female **(B)** volunteers.

This study ultimately selected 23 HUA volunteers, including 15 males and eight females. After taking DH, the UA levels of volunteers all decreased (Figure 9). This result indicates that DH can effectively reduce UA levels in patients with HUA.

## 4 Discussion

HUA is a chronic metabolic disease caused by purine metabolism disorders, mainly due to reduced or increased excretion of UA. In addition, consuming excessive foods high in purine may also trigger the onset of HUA (20). This study constructed a HUA mouse model by administering PIO and HX via gavage, which increased the intake of high purine foods and reduced uric acid excretion. The results showed that after oral administration of PIO + HX, the UA in the serum of mice significantly increased, which is consistent with the symptoms of HUA. These results indicate that the HUA modeling in this study was successful. After administering fresh DH powder orally to HUA mice, the UA level of HUA mice significantly decreased. These research results indicate that DH has a therapeutic effect on HUA.

HUA can cause kidney damage, as the kidneys are the main organs for metabolizing UA. Therefore, after kidney damage, UA metabolism disorders can occur, further exacerbating HUA (21). Cr is a toxin produced by muscle metabolism, mainly cleared by the kidneys. When the kidneys are damaged, the serum Cr content will significantly increase, so Cr is one of the important indicators for evaluating kidney function (22). In this study, we found a significant increase in Cr content in the serum of HUA mice, and HE staining results also showed damage to the kidney tissue of HUA mice. DH can significantly reduce Cr content and alleviate kidney tissue damage. BUN is a nitrogen-containing compound in plasma that is excreted from the body after filtration by the glomerulus, excluding proteins. When renal dysfunction is decompensated, BUN will increase. So BUN is an important indicator for judging glomerular filtration function (23). In this study, we observed a significant increase in BUN levels in the serum of HUA mice, and PAS staining results also showed glomerular damage in HUA mice. DH treatment significantly reduces BUN levels and alleviates glomerular damage. These results all indicate that DH can alleviate kidney damage caused by HUA.

XOD plays an important role in the occurrence and development of HUA. XOD is an enzyme with low specificity that can catalyze the conversion of HX to xanthine, which in turn generates UA, as well as directly catalyze the conversion of xanthine to UA (24). Numerous studies have shown that inhibiting the activity of XOD can effectively reduce the content of UA in serum, and some XOD inhibitors have also been used to treat HUA, such as 5-formyluracil, heavy metal ions, imidazole, etc. Although these XOD inhibitors have some effectiveness in treating HUA, there are also some side effects such as urate deposition, indigestion, rash, liver dysfunction, and muscle pain (25, 26). This study found that the activity of XOD in the serum of HUA mice was significantly increased, while DH treatment could significantly reduce the activity of XOD in the serum of HUA mice. This result suggests that DH may be a natural XOD inhibitor.

Metabolomics is a research method that imitates the research ideas of genomics and proteomics, quantitatively analyzes all metabolites in an organism, and searches for the relative relationship between metabolites and physiological and pathological changes. It is an integral part of systems biology (27). Metabolomics can study drug therapy for diseases at the overall metabolic level and has been widely used to investigate the pharmacological mechanisms of traditional Chinese medicine (28, 29). This study conducted serum metabolomics analysis on various groups of mice and found that DH can reverse the disorders of pyrimidine metabolism, histidine metabolism, and riboflavin metabolism caused by HUA. Pyridine metabolism, histidine metabolism, and riboflavin metabolism have been found to be involved in purine metabolism and play important roles in the pathogenesis of HUA. These research findings suggest that DH may treat HUA by improving pyrimidine metabolism, histidine metabolism, and riboflavin metabolism.

This study has demonstrated the therapeutic effect of DH on HUA at the animal level, but its clinical efficacy cannot be determined yet. In order to study the clinical efficacy of DH, this study recruited 40 volunteers. After detecting UA levels, 23 high uric acid volunteers were selected, including 15 males and 8 females. As a result, it was found that the UA levels of volunteers decreased after taking fresh DH powder. This result further indicates that DH has therapeutic effects on HUA.

There are several limitations to this study. ① Fresh DH powder is a mixture, and the active ingredients for anti-HUA effects are not yet clear. ② This study lacked research on the mechanism of the anti-HUA effect of fresh DH powder. ③ This study revealed decreased UA levels in HUA volunteers after they drank fresh DH powder, lacked a blank control group, and cannot directly prove that fresh DH powder has anti-HUA effects. Our team will gradually address these issues in future research.

## 5 Conclusion

In conclusion, DH has a preventive and therapeutic effect on HUA, and its uric acid lowering effect may be related to its improvement of pyrimidine metabolism, histidine metabolism, and riboflavin metabolism disorders. Compared to drugs, DH belongs to the category of medicinal food homology, with extremely low toxicity and high safety. And this study used DH fresh medicine as raw material, and the entire preparation process was low-temperature treated, which can largely preserve the pharmacological activity of DH. Compared with traditional Chinese medicine decoction pieces, it has better pharmacological activity. These provide strong evidence for developing DH into products with anti HUA effects. Based on the results of this study, our team has designed two functional food products with DH as the main ingredient for anti HUA effects.

## Data Availability

The original contributions presented in the study are included in the article/supplementary material, further inquiries can be directed to the corresponding author.
